# Stakeholder Pressure and Green Innovation: A Meta‐Analytic Exploration of Industry and Regional Variations

**DOI:** 10.1002/brb3.70840

**Published:** 2025-09-09

**Authors:** Hasan Emin Gurler, Ahmet Kaya, Ayşe Nur Soysal Bilmiş, Yakup Durmaz

**Affiliations:** ^1^ Department of International Trade and Logistics, Faculty of Economics and Administrative Sciences Kilis 7 Aralık University Kilis Turkey; ^2^ Department of Finance and Banking, Faculty of Applied Sciences Akdeniz University Antalya Turkey; ^3^ Department of Marketing and Advertising, Korkuteli Vocational School Akdeniz University Antalya Turkey; ^4^ Faculty of Economics and Administrative Sciences Kilis 7 Aralık University Kilis Turkey

**Keywords:** green innovation, industry context, meta‐analysis, regional disparities, stakeholder pressure

## Abstract

**Purpose:**

Stakeholder pressure is widely recognized as a key driver of green innovation. However, existing research often overlooks how this relationship may differ across various industry settings and national contexts. Understanding these contextual differences is essential for businesses aiming to implement effective and responsive environmental strategies.

**Methods:**

This study synthesizes findings from 23 independent investigations conducted across multiple industries and countries. The analysis aims to uncover patterns in how stakeholder pressure influences green innovation, with a particular focus on variations between sectors and regional settings.

**Findings:**

The influence of stakeholder pressure on green innovation is generally positive but varies significantly by context. In manufacturing industries, the relationship is weaker, likely due to technical, operational, or financial constraints that hinder firms from fully meeting stakeholder expectations. The effect is also less pronounced in China, potentially due to country‐specific stakeholder norms, regulatory frameworks, or socio‐economic factors that shape corporate environmental responses.

**Conclusions:**

Both industry characteristics and national contexts significantly shape the effectiveness of stakeholder‐driven green innovation. Firms need to consider these contextual nuances when developing environmental strategies. Tailoring approaches to fit specific industrial and regional conditions can improve alignment with stakeholder expectations and enhance the success of green initiatives.

## Graphical abstract

This systematic review synthesizes organizational‐level studies on the link between stakeholder pressure and green innovation. From an initial sample of 1,745 records, 23 empirical studies met the eligibility criteria. Results highlight that stakeholder demands act as a significant driver of sustainable and eco‐innovations, suggesting that strengthening stakeholder engagement is a key pathway toward advancing environmental innovation.

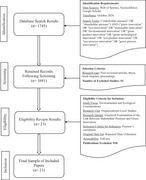

## 1 Introduction

Over the past few decades, increasing political, social, and economic pressures related to environmental issues have led companies to place greater emphasis on these concerns in their strategic and operational planning (Sarkis et al. 2010, 163). The growing global emphasis on environmental sustainability has compelled organizations to incorporate environmental considerations into their strategic and operational activities. Stakeholder theory highlights that businesses are significantly influenced by diverse groups such as governments, investors, customers, and NGOs, whose pressures shape corporate environmental practices (Gallego‐Alvarez et al. 2017, 808; Meixell and Luoma 2015, 70). Notably, these pressures are not uniform across industries; firms in dynamic sectors often encounter higher levels of stakeholder demands compared to those in static industries (Betts et al. 2015, 283). Furthermore, studies emphasize the pivotal role of managerial values and stakeholder perceptions in driving proactive environmental practices, particularly in areas like logistics and supply chain management (González‐Benito and González‐Benito 2006, 1354). Successfully addressing these external pressures requires organizations to align their environmental strategies with stakeholder expectations, ensuring both environmental accountability and operational resilience (Helmig et al. 2016, 151).

Stakeholder pressure has become a significant driver for green innovation, urging firms to integrate sustainable practices into their operations to meet environmental and social expectations (Ma and Chen 2023, 2). Green innovation refers to the development and implementation of eco‐friendly products, processes, and systems that aim to reduce environmental harm while improving economic outcomes (Zhang et al. 2020, 3). This innovation not only addresses regulatory and customer pressures but also enhances resource efficiency and creates competitive advantages through sustainability (Singh et al. 2022, 500). By responding to stakeholder demands and leveraging green knowledge exchange, organizations can enhance both their environmental and financial performance, underscoring the strategic importance of aligning corporate goals with sustainable innovation practices (Jiang et al. 2024, 3).

Previous studies have emphasized the pivotal role of stakeholder pressure in fostering green innovation, with varying findings on the nature of this relationship (Cheng, Zhang et al. 2024; Ma and Chen 2023; Jiang et al. 2024). Despite this, the literature lacks a comprehensive exploration of how contextual factors, such as country and industry, moderate this relationship. This study aims to address this critical gap by investigating the influence of these moderators, offering a more nuanced understanding of the stakeholder pressure‐green innovation dynamic. Moreover, it seeks to provide robust evidence on the generalizability of this relationship across diverse national and industry contexts, thereby advancing theoretical frameworks and informing practical applications.

This research significantly enriches current academic debates by examining how stakeholder pressure influences green innovation, paying particular attention to how this interplay changes depending on both industry and national contexts. Drawing on meta‐analytic methods and synthesizing insights from 23 studies across various sectors and geographical regions, it uncovers the multifaceted nature of these relationships. Notably, the analysis pinpoints pronounced differences within the manufacturing sector and in the context of China. By looking closely at industry‐specific obstacles and regional nuances, the study fills a crucial gap in our understanding of how pressure from stakeholders can effectively spur eco‐friendly innovation. It also extends the conversation within stakeholder theory, illustrating how companies can smartly align their environmental efforts with what stakeholders expect, all while circumventing the structural and operational hurdles that often hinder progress. On a practical level, these findings will be of genuine use to policymakers and professionals who need to consider each sector's unique constraints and local conditions when crafting policies and initiatives to drive sustainable innovation. Taken together, the blend of strong theoretical insights and pragmatic recommendations offers a sturdy platform for future inquiries, encouraging further research that delves into under‐examined geographic areas, industries, and long‐term patterns in green innovation.

## 2 Literature

### 2.1 Stakeholder Pressure and Green Innovation

Stakeholder pressure is a significant force that determines the level of accountability a company maintains while conducting its activities and influences its decisions. Stakeholder pressure refers to the demands originating from customers, governments, employees, and other external factors. Specifically, with the tightening of environmental regulations and the growing environmental awareness in society, pressures on companies to adopt green practices have increased (Singh et al. 2022, 503). Governments, through regulatory bodies, examine the environmental performance of companies, which drives businesses to restructure their dynamic capabilities and shift toward sustainable practices (Jin et al. 2024, 2).

Environmentally friendly products and services demanded by customers and society lead firms to develop green strategies. At the same time, these pressures from stakeholders force companies to restructure their existing resources and improve their environmental performance. Firms facing stakeholder pressure aim to achieve sustainability goals by developing green dynamic capabilities (Tang et al. 2023, 1044). In this context, stakeholder pressure becomes a significant factor triggering firms to adopt green innovation strategies (GIS). Green innovation refers to the development of new products, processes, technologies, and management systems by firms to provide cost‐effective solutions to environmental problems. These innovations, also defined as eco‐friendly practices, aim to enhance energy and resource savings while reducing waste and emissions (Chen et al. 2018, 304). Green innovation particularly encompasses activities such as reducing the amount of raw materials used in production processes, facilitating the recycling of products after use, and ensuring energy efficiency (Khanra et al. 2022, 1396).

GIS help firms improve their environmental performance while also assisting them in complying with strict environmental regulations. Such innovations not only create new market opportunities for businesses but also provide competitive advantages that strengthen corporate image and enhance customer loyalty (Singh et al. 2022, 501). However, green innovation often requires high costs and long‐term investments. Therefore, it is crucial for firms to view green innovation as a strategic issue and to advance in this area with the support of stakeholders (He and Jiang 2019, 1341).

Stakeholder pressure is a driving force behind firms' engagement in green innovation activities. The increasing expectations for environmental and social responsibility encourage firms to develop new products, processes, and management systems based on green innovation (Singh et al. 2022, 504). Stakeholder pressure leads firms to use eco‐friendly materials, adopt energy‐saving technologies, and produce innovative solutions that reduce waste (Chen et al. 2018, 304). Moreover, governmental pressures and consumer demands help firms achieve competitive advantages through sustainable practices and increase their market share (Zhao et al. 2023, 199). Consequently, stakeholder pressure shapes firms' GIS, enabling them to improve their environmental performance. This pressure lays the groundwork for firms to achieve sustainable competitive advantages while simultaneously creating societal and environmental benefits (Tang et al. 2023, 1043). Drawing on the previously mentioned literature, the first hypothesis is formulated as follows:

**H1**: Stakeholder pressure has a positive impact on green innovation, as rising stakeholder pressure results in greater levels of green innovation.

### 2.2 Internal Capabilities and Green Innovation

While stakeholder pressure plays a significant role in motivating firms to adopt green practices, internal capabilities such as organizational learning, dynamic capabilities, and research and development (R&D) investments are equally crucial for transforming green strategies into competitive outcomes. GI serves as a mediating mechanism linking these internal capabilities to sustainable performance. As shown by Nguyen et al. (2023), green dynamic capabilities and green creativity significantly enhance competitive advantage, with GI acting as a key mediator in this process. This underscores the importance of firms’ internal learning and adaptive capacity to drive sustainable success.

Organizational learning is particularly central to this transformation. Tu and Wu (2021) found that “green innovation was positively related to enterprises’ competitive advantage, and this process was mediated by organizational learning,” suggesting that without a learning‐oriented organizational culture, firms may struggle to realize the benefits of green initiatives. In a similar vein, Özgül and Zehir (2023) highlight green organizational learning capability as a bridge between green leadership and competitive success. Their framework integrates green absorptive and transformative capabilities, enabling firms to process, internalize, and apply green knowledge effectively. In this context, cooperation with external partners—including suppliers, universities, and technology centers—has been shown to significantly enhance green innovation efforts, particularly when internal R&D alone is insufficient (Sánchez‐Sellero and Bataineh 2022).

Moreover, R&D efforts amplify the impact of green innovation on competitive advantage. Bataineh et al. (2024a,b) demonstrate that R&D expenditures significantly strengthen the positive effects of GI on competitive performance, especially in terms of product quality, differentiation, and market share. Regulatory mechanisms can also stimulate internal innovation capabilities. Similarly, green R&D investments have been found to reduce carbon emissions while simultaneously improving firm‐level financial performance, highlighting the dual environmental and economic benefits of innovation‐oriented capabilities (Lee and Min 2015). For example, Zhou (2024) finds that environmental policies such as China's Five‐Year Plans drive high‐polluting firms to invest in R&D, thereby enhancing GI quality and gaining a strategic edge. These findings suggest that the internal capability base of a firm—including its learning culture, innovation processes, and R&D investment—is a critical determinant of how effectively it can convert green pressures and opportunities into long‐term competitive advantage. Moreover, empirical evidence indicates that R&D is a critical enabler for renewable energy‐related green technology innovation, especially when supported by digital infrastructure (Dogan et al. 2025). From a broader perspective, upgrading the technological innovation value chain has been shown to foster green transformation by increasing the efficiency and productivity of innovation systems, especially in high‐tech industries (Cheng, Wang et al. 2024).

### 2.3 Green Innovation and Competitive Advantage

Internal capabilities, however, do not operate in isolation; rather, their effectiveness is amplified when integrated with broader strategic approaches to green innovation. In this regard, GI has increasingly been recognized as a strategic resource that contributes directly to firms’ sustainable competitive advantage. As Barforoush et al. (2021) emphasize, GI encompassing organizational, technological, stakeholder, and policy dimensions plays a critical role in enabling firms to develop competitive advantages by enhancing eco‐efficiency and responding proactively to environmental regulations and societal expectations. Also, Ge et al. (2018) empirically demonstrate that the successful implementation of GIS leads to sustainable competitive advantages through the mediating effect of dynamic capabilities. Their findings suggest that while GIS may incur initial costs, its effective deployment helps firms align internal capabilities with external environmental uncertainty, ultimately reinforcing market competitiveness. Organizational innovation has also been identified as a fundamental driver of green innovation, especially when firms adapt their internal structures to align with updated environmental regulations and external technological shifts (Bataineh et al. 2024b).

Similarly, Van et al. (2025) show that green innovation indirectly enhances sustainable competitive advantage through improved sustainability reporting, especially when supported by environmental management accounting and green absorptive capacity. These dynamic capabilities allow firms to transform green practices into measurable, reportable outcomes that resonate with stakeholder demands and improve market position. Also, Hayat and Qingyu (2024) also find that GIS, particularly in product, process, and service dimensions, significantly boosts a firm's green innovative competitive advantage, which in turn enhances long‐term innovative performance and competitiveness. Their results reinforce the relevance of green innovation in modern strategic management, especially when integrated with CSR initiatives. Furthermore, foreign direct investment significantly supports innovation outcomes such as patents and product development, particularly when firms invest in R&D and foster external collaborations that enhance absorptive capacity (Sánchez‐Sellero and Bataineh 2024).

Additionally, export activity has been found to indirectly promote green technology innovation through increased access to government subsidies and heightened environmental awareness, particularly among SMEs (Yang et al. 2024). In a similar vein, firms leveraging advanced technologies and intellectual property protections—especially patents—benefit more strongly from export‐driven green innovation initiatives (Bataineh and Sánchez‐Sellero 2025).

### 2.4 Moderating Role of Country and Industry

The relationship between stakeholder pressure and green innovation is likely to vary depending on different factors. In this context, the study examined whether this effect differs across countries and sectors by analyzing the moderating role of these two variables.

In the realm of green innovation, stringent regulatory pressures necessitate greater focus on achieving greening objectives, which may evolve into a central orientation at the firm level (He and Su 2022, 6). In response to these pressures, companies—particularly those operating in emerging markets such as China—often adopt existing production standards, like ISO certifications, to implement green process innovations (Zhang and Zhu 2019, 1016). At the same time, rising environmental demands from both international and domestic consumers are prompting many Chinese manufacturing companies to reevaluate their strategies and incorporate environmental considerations into their organizational policies and operations. For instance, an increasing number of Chinese firms are pursuing environmental certifications, such as ISO 14001, to address external pressures related to sustainability (Zhang and Zhu 2019, 1017).

In these emerging economies, regulatory authorities play a critical role by introducing measures such as subsidies, taxation, and the establishment of laws, administrative strategies, and informal guidelines. These measures aim to mitigate the negative impacts of environmental challenges on the collaborative growth of businesses, markets, and society. As a result, regulatory pressures strengthen firms' operational and support systems, encouraging the adoption of flexible and adaptable organizational structures. This, in turn, fosters the development and implementation of green technologies (He and Su 2022, 5). Ultimately, such rigorous regulatory pressures drive companies to voluntarily adopt globally recognized environmental standards within their industries, supporting a broader transition toward sustainability (He and Su 2022, 6). Building on the insights from the aforementioned literature, the second hypothesis is proposed as follows:

**H2**: In China, stakeholder pressure has a greater influence on green innovation than it does in other countries.

Regulatory pressures often pose significant challenges for companies, as noncompliance can result in severe penalties that threaten their survival. Consequently, firms under intense regulatory scrutiny tend to focus on exploitation strategies, enabling them to quickly adopt green process innovations by optimizing their production methods (Zhang and Zhu 2019, 1016). Manufacturing enterprises, in particular, face substantial oversight due to environmental challenges, with their actions being closely monitored by stakeholders such as government bodies, local communities, investors, and consumers. This reflects the growing emphasis on sustainable practices in industrial operations (Rui and Lu 2021, 76). As the adoption of green practices in manufacturing gains momentum, it has become a critical concern for both manufacturers and researchers to identify and prioritize the key enablers of green innovation within organizations (Gupta and Barua 2018, 9556). In parallel, the integration of advanced digital manufacturing technologies and precision equipment has unlocked new opportunities for innovation in production and delivery. These technologies not only enhance quality and value but also shorten development timelines and support the transition to green manufacturing practices (Shahzad et al. 2022, 1).

**H3**: The impact of stakeholder pressure on green innovation is more significant in manufacturing firms compared to other industries.

## 3 Research Methods

### 3.1 Data Collection Process

**FIGURE 1:**
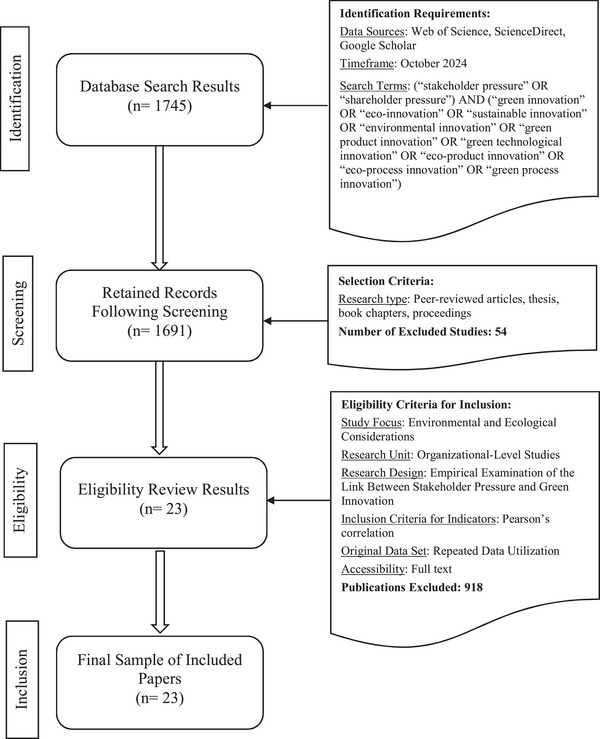
The PRISMA framework.

This study adopts the PRISMA (Preferred Reporting Items for Systematic Reviews and Meta‐Analyses) framework to systematically delineate the sample, ensuring a rigorous and transparent selection process. The PRISMA framework, first introduced by Moher et al. (2009) and subsequently updated as PRISMA‐2020 (Page et al. 2021), delineates essential reporting standards for authors engaged in systematic reviews of healthcare interventions. Endorsed by prominent academic journals, PRISMA has been widely cited across the literature. Empirical evidence indicates that its implementation has led to enhanced transparency and rigor in the reporting practices of clinical research reviews, thereby elevating the overall quality of evidence synthesis in this domain (O'Dea et al. 2021, 1696). Compared to other systematic review methods (e.g., traditional narrative reviews), PRISMA was preferred because of its transparency in the screening process and reduced risk of bias (Tricco et al. 2018, 469). In particular, standardization of source screening and exclusion criteria increases reproducibility in meta‐analyses (Page et al. 2021, 2‐3). The PRISMA framework encompasses four distinct stages: identification, screening, eligibility assessment, and inclusion (Moher et al. 2009). A more detailed explanation of these stages is provided in Figure 1.

Initially, key elements such as relevant keywords, appropriate databases, and the search timeframe are established as part of the identification process. This study has successfully identified and classified two distinct groups of keywords: (“stakeholder pressure,” “shareholder pressure”) and (“green innovation,” “eco‐innovation,” “sustainable innovation,” “environmental innovation,” “green product innovation,” “green technological innovation,” “eco‐product innovation,” “eco‐process innovation,” “green process innovation”). A comprehensive search was performed across the Google Scholar, Web of Science, and ScienceDirect databases, utilizing specific keywords to identify relevant scholarly articles. Moreover, a systematic review of the indexed documents within these databases was concluded by the end of October 2024. A comprehensive set of 2540 papers was sourced from Google Scholar, 47 from Web of Science, and 338 from ScienceDirect. After systematically merging these datasets and removing duplicate entries, the resulting corpus consisted of 1745 distinct and non‐redundant papers.

Secondly, the screening process employs a stringent quality criterion to uphold the academic rigor and validity of the research. Specifically, the sample is comprised exclusively of peer‐reviewed journal articles, theses, conference proceedings, and book chapters. As a result, a final sample of 1691 papers is derived, ensuring a robust basis for analysis.

Third, the papers are subjected to a comprehensive evaluation based on six explicit eligibility criteria: (1) The study must predominantly focus on environmental and ecological considerations. (2) Stakeholder pressure and green innovation are defined as firm‐level variables. Consequently, only studies conducted at the firm level are eligible for inclusion. (3) The research must adopt a quantitative approach, utilizing clearly defined constructs and measurement tools, while concurrently addressing both stakeholder pressure and green innovation. Studies with ambiguous construct definitions, mathematical modeling‐based research, or case studies are excluded. (4) The research must calculate the Pearson correlation between stakeholder pressure and green innovation. Papers reporting only *β* coefficients are not considered, as meta‐analytic results derived from Pearson's correlations provide a more precise and reliable measure. Pearson correlation is considered a critical standard in meta‐analyses as it standardizes and makes comparable studies on different scales (Hunter and Schmidt 2004, 45‐47). Since *β* coefficients are model specific, they are dependent on sample and control variables (Peterson and Brown 2005, 175‐176). (5) In many cases, the same studies are indexed across multiple databases. To address this, duplicate entries are systematically identified and removed, ensuring that only a single instance of each study, selected from one database, is retained for analysis. (6) This study excluded papers with inaccessible full texts, given that obtaining the Pearson correlation coefficient is fundamental to the meta‐analysis methodology employed. During this phase of the research, six meticulously formulated criteria were rigorously employed, leading to the exclusion of 918 studies from further analysis.

Following the completion of the PRISMA process, a rigorously validated sample of 23 studies was finalized. Results from 23 studies are considered methodologically adequate for Pearson's r‐based meta‐analyses. For example, Field and Gillett (2010, 674) stated that 15‐20 studies is the minimum threshold for stabilization of effect sizes. The earliest study in this collection was published in 2015, whereas 19 studies appeared between 2020 and 2024. This temporal distribution highlights the escalating scholarly attention and growing recognition of the stakeholder pressure‐green innovation relationship as a critical and emerging area of contemporary research.

### 3.2 Evaluation Measures

The research framework incorporated three primary categories of variables, namely independent variables, dependent variables, and moderating variables, to comprehensively examine the relationships under investigation.

#### 3.2.1 Independent Variable

The independent variable is conceptualized through two primary constructs: “stakeholder pressure” and “shareholder pressure.”

#### 3.2.2 Dependent Variable

Green innovation encompasses a broad spectrum of innovative strategies designed to address environmental challenges effectively. These strategies include a variety of concepts, such as “green innovation,” “eco‐innovation,” “sustainable innovation,” “environmental innovation,” “green product innovation,” “green technological innovation,” “eco‐product innovation,” “eco‐process innovation,” and “green process innovation.”

#### 3.2.3 Moderator Variables

This meta‐analysis identifies industry and country as two pivotal moderators, emphasizing their significance in contextualizing the dynamic relationship between stakeholder pressure and green innovation. For analytical purposes, studies exclusively centered on the manufacturing sector were assigned the code “1.” Conversely, research incorporating multiple sectors within a single study was classified as “2” (Mixed). Studies that concentrated solely on a distinct sector were labeled as “3” and grouped under the category “Other.” Finally, the variable ‘country’ was coded as 1 for China and 2 for Other, with the ‘Other’ category including Turkey, Ghana, Vietnam, Taiwan, India, Japan, the United Arab Emirates, Indonesia, and Pakistan.

### 3.3 Impact of Publication Bias

**FIGURE 2:**
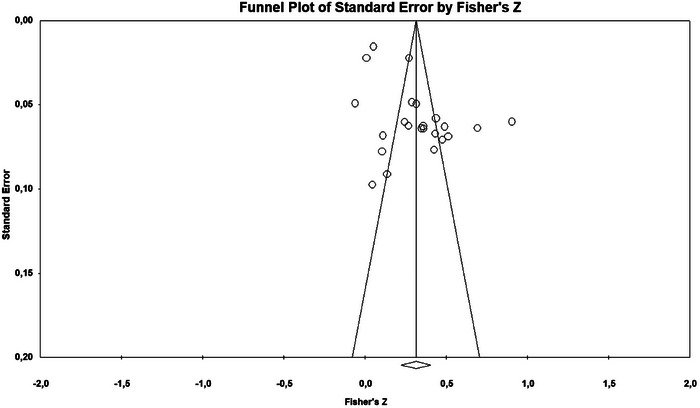
The funnel plot.

Rosenthal's (1991) “fail‐safe N” (NFS) estimates the number of unpublished null studies needed to nullify the significance of meta‐analysis results. While NFS is a tool to evaluate result confidence, recent studies suggest it may differ significantly from alternative methods estimating unreported studies. These alternatives rely on reported *P*‐values and assume the null hypothesis is true (Thornton and Lee 2000, 212). Publication bias may be present when the fail‐safe *N* value falls below the critical threshold of 5k + 10 (where *k* represents the number of included studies). This criterion serves as a robust indicator of potential file‐drawer effects, wherein statistically non‐significant findings remain unpublished. Consequently, a fail‐safe *N* value exceeding this threshold provides empirical evidence that the observed meta‐analytic effect is resistant to potential publication bias (Infurna et al. 2016, 51). Our analysis yielded a fail‐safe N of 4029, substantially above this threshold, indicating minimal risk of publication bias. Furthermore, the rank correlation test by Begg and Mazumdar produced an insignificant result (p > 0.1). A symmetrical funnel plot, depicted in Figure 2, corroborates these findings, affirming that publication bias does not meaningfully impact the study's outcomes.

### 3.4 Procedures for Meta‐Analysis

In the context of multivariate effect sizes, sampling errors are typically conditionally correlated due to the reliance on the same participant pool for calculating multiple effect sizes. Additionally, a single study may report several effect sizes from independent samples, all targeting the same construct pertinent to the research inquiry. Consequently, aggregating these effect sizes into a single composite effect size is a valid approach for analysis (Cheung 2019, 389). Corrected correlations are subsequently transformed into Fisher's *z*‐values to minimize the effects of sampling variance, ensuring greater statistical robustness.

Assessing heterogeneity in meta‐analysis plays a pivotal role, as it influences the meta‐analyst's decision regarding the appropriate statistical model—fixed‐effects or random‐effects—to apply. Traditionally, the Q test has served as the primary approach to evaluating the heterogeneity hypothesis. However, the I^2^ statistic, alongside its confidence interval, offers an enhanced framework to both estimate the magnitude of heterogeneity and determine its statistical significance, thereby complementing or refining traditional methods (Huedo‐Medina et al. 2006, 203).

## 4 Empirical Findings

### 4.1 Effect Size

Meta‐analysis typically relies on two statistical models: the fixed‐effect model and the random‐effects model. The fixed‐effect model assumes a single true effect size shared by all studies, with variations attributed solely to sampling error, whereas the random‐effects model permits true effect sizes to vary due to differences in study conditions, such as participant demographics or intervention intensity. This distinction highlights how variability across studies can influence underlying effect sizes (Borenstein et al. 2010, 97). The adoption of the random effects model is warranted due to the significant heterogeneity demonstrated by the high *Q* statistic and I^2^ values. Therefore, it is deemed the most suitable approach for the analysis (Borenstein et al. 2010, 106). The random effects model analyzes the relationship between stakeholder pressure and green innovation by addressing heterogeneity in effect sizes across studies. This methodology is crucial to account for study‐specific factors that uniquely shape the observed outcomes.

**FIGURE 3:**
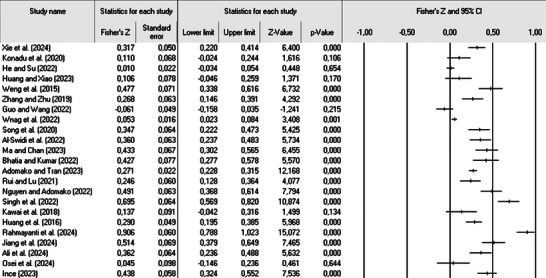
The forest plot (Xie et al., 2024; Konadu et al., 2020; He & Su, 2022; Huang & Xiao, 2023; Weng et al., 2015; Zhang & Zhu, 2019; Guo & Wang, 2022; Wang et al., 2022; Song et al., 2020; Al‐Swidi et al., 2022; Ma & Chan, 2023; Bhatia & Kumar, 2022; Adomako & Tran, 2023; Rui & Lu, 2021; Nguyen & Adomako, 2022; Singh et al., 2022; Kawai et al., 2018; Huang et al., 2016; Rahmayanti et al., 2024; Jiang et al., 2024; Ali et al., 2024; Osei et al., 2024; İnce, 2023).

This study involved estimating 23 effect sizes, which are summarized in the forest plot presented in Figure 3. In meta‐analyses, 20‐30 studies are sufficient for reliable estimation of average effect sizes (Borenstein et al. 2009, 112). The plot includes key information such as the authors' names, Fisher's *Z* values, publication years, and the relative weight assigned to each study. The effect sizes are predominantly clustered around a mean value of 0.314. Based on Cohen's (1988) guidelines for effect size classification, the correlation coefficient and sample size calculations indicate a moderate and positive relationship between stakeholder pressure and green innovation. Consequently, H_1_ is supported.

The *Q* statistic, with a value of 527.094 and 22 degrees of freedom (df), reveals a statistically significant result at the 95% confidence level (*p* = 0.000). To assess the heterogeneity of the data, the I^2^ statistic is also considered, which in this study is 95.826. These elevated values of *Q* and I^2^ suggest considerable heterogeneity in the relationship between stakeholder pressure and green innovation across the included studies, highlighting the necessity for further investigation to better understand this variability. Given the heterogeneity of the dataset, the ANOVA methodology was employed (Lipsey and Wilson 2001). Following Hunter and Schmidt's (2004, 401) recommendations, we examined between‐study heterogeneity to determine whether moderator analyses were warranted. When the percentage of variance attributable to sampling error falls below 75%, this indicates substantial heterogeneity that may be explained by moderator variables (Infurna et al. 2016, 51). Thus, the analysis focused on two categorical variables: industry and country. As a result, subgroup ANOVA analyses were performed, and the findings are detailed in the subsequent sections.

### 4.2 Industry‐Related Effects

The study argues that the relationship between shareholder pressure and green innovation differs across industries, particularly within the manufacturing sector. To examine this, studies focusing on the manufacturing sector were coded as 1, while those incorporating multiple sectors as samples in the same study were coded as 2 (Mixed). Studies that focused solely on a single sector (e.g., mining, tourism) were coded as 3, under the “Other” category. This classification was based on prior literature indicating that manufacturing sectors, due to their higher resource intensity and environmental impacts, experience stronger stakeholder pressure effects on green innovation adoption compared to other industries (Rui and Lu 2021, 76; Gupta and Barua 2018, 9556).

The ANOVA method was applied to assess the influence of categorical variables, with both fixed‐effects and mixed‐effects models employed to enable subgroup analyses. Borenstein et al. (2009) emphasized the critical role of evaluating the significance of the Q_TOTAL_ statistic within the fixed‐effects model. A significant result from this test necessitates further investigation of the mixed‐effects model outcomes. Consistent with the fixed‐effects model findings, all *Q* values were found to be statistically significant (*Q*_MANUFACTURING_ = 74.599; *Q*_MIXED_ = 57.351; *Q*_OTHER_ = 109.963; *Q*_W_ = 241.913; *Q*_B_ = 285.182; *Q*_TOTAL_ = 527.094). Since QTOTAL indicated statistical significance, a mixed‐effects model was utilized to examine potential effects, and the corresponding results are summarized in Table 1.

**TABLE 1:** ANOVA results by industry.

Subgroup	# study	Effect Size	Lower limit	Upper limit	Z‐value	*p*
Manufacturing	14	0.327	0.253	0.398	8.189	0.000
Mixed	4	0.110	−0.016	0.232	1.717	0.086
Other	5	0.387	0.165	0.572	3.311	0.001
Overall	23	0.275	0.213	0.334	8.415	0.000

*Note*: Q_B_ = 10.160, df = 2, *p* = 0.006; Manufacturing = 1; Mixed = 2; Other = 3.

The results presented in Table 1 highlight notable differences in effect sizes across the various industry subgroups analyzed through ANOVA. The manufacturing subgroup demonstrates a strong and statistically significant effect, indicating a clear impact within this sector. On the other hand, the mixed subgroup shows a smaller effect that is not statistically significant, suggesting that the findings in this category may not be as robust. The other subgroup exhibits a moderate but significant effect, indicating that industries outside of manufacturing and mixed also experience a noteworthy impact. Overall, the analysis of all subgroups combined reveals a significant effect across the entire sample. Furthermore, the *Q* statistic (*Q*_B_ = 10.160, *p* = 0.006) suggests that the variation in effect sizes between industries is statistically significant, supporting the conclusion that industry type plays a crucial role in determining the observed effects. As a result, H_2_ is not supported.

### 4.3 Country‐Specific Effects

A subgroup analysis was carried out to assess potential variations in effect size across countries. The variable “country” was dichotomized into two categories: “China” and “Other,” based on the geographical origin of the study sample. The “Other” category includes Turkey, Ghana, Vietnam, Taiwan, India, Japan, the United Arab Emirates, Indonesia, and Pakistan. This classification was based on prior literature suggesting that China's unique regulatory environment (e.g., stringent policies, subsidies) leads to stronger stakeholder pressure effects compared to other emerging economies (He and Su 2022, 5; Zhang and Zhu 2019, 1016).

The *Q* values for the examined categories were determined as follows: *Q*_CHINA_ = 159.252, *Q*_OTHER_ = 164.733, *Q*_W_ = 323.986, *Q*_B_ = 203.109, and *Q*_TOTAL_ = 527.094. The significant *Q*_TOTAL_ value warranted further exploration of potential effects through a mixed‐effects model, the results of which are detailed in Table 2.

**TABLE 2:** ANOVA results by country.

Subgroup	# study	Effect size	Lower limit	Upper limit	Z‐value	*p*
China	11	0.220	0.126	0.310	4.511	0.000
Other	12	0.378	0.264	0.482	6.115	0.000
Overall	23	0.280	0.207	0.349	7.293	0.000

*Note*: Q_B_ = 4.549, df = 1, *p* = 0.033; China = 1; Other = 2.

Table 2 summarizes the ANOVA results for subgroup analyses by country. The effect size for studies conducted in China is moderate and statistically significant, indicating a consistent effect across these studies. Similarly, studies from other countries show a larger, statistically significant effect size, suggesting a stronger impact in this subgroup. Overall, the combined effect size is significant, reinforcing the reliability of the observed patterns across all studies. Notably, the *Q*_B_ statistic reveals a significant difference between the two subgroups, suggesting that the effect sizes vary depending on the geographical context. This emphasizes the potential influence of country‐specific factors on the observed outcomes. Consequently, the findings do not provide support for hypothesis H_3_.

## 5 Discussion

This study aimed to investigate the relationship between stakeholder pressure and green innovation, with a particular focus on the moderating effects of industry and country contexts. A total of 23 studies were analyzed using meta‐analytic techniques, encompassing a wide range of industries and geographical locations. The findings reveal significant heterogeneity across the studies, with notable variations in effect sizes based on industry type and country‐specific factors. Subgroup analyses indicated that while stakeholder pressure generally has a positive impact on green innovation, the strength of this relationship varies considerably, particularly in the manufacturing sector and in studies conducted in China. These results provide important insights into the contextual dynamics influencing green innovation and offer a basis for further exploration of sectoral and regional differences.

The relationship between stakeholder pressure and green innovation in the manufacturing sector presents a multifaceted challenge, one that scholars have approached from various angles. On one hand, the industry's pronounced ecological footprint intensifies outside scrutiny and compels a reassessment of traditional production models. According to Jiao et al. (2020), the substantial consumption of natural resources and generation of industrial waste have placed manufacturers under considerable regulatory, social, and economic pressure to adopt sustainable practices. Chen et al. (2020) similarly emphasize how escalating environmental risks—encompassing issues such as greenhouse gas emissions, resource depletion, and water contamination—are increasingly prompting manufacturers to prioritize environmentally responsible strategies that mitigate their ecological impact. Meanwhile, Jakhar et al. (2020) call attention to the necessity of achieving a delicate equilibrium: pairing the immediate gains of exploitative innovation with the forward‐looking promise of exploratory innovation. Such a balance enables manufacturing firms to catalyze sustainability, blending efficiency with a long‐term commitment to green transformation. Collectively, these insights underscore that while the manufacturing sector is subject to strong external demands for ecological stewardship, it also holds significant latent potential to leverage this pressure into genuine environmental progress.

While stakeholder pressure has undoubtedly encouraged greener approaches, the manufacturing sector still grapples with distinctive obstacles that frequently impede the full realization of such innovations. Bello‐Pintado et al. (2023) illustrate this tension: even as environmental concerns mount, many manufacturers persist in reacting to sustainability issues only after the fact, thereby diminishing the influence that stakeholders might otherwise exert. Likewise, Azam et al. (2021) highlight the tangible and intangible costs tied to environmental improvements—substantial financial investments, lengthy roll‐out periods, and inherent uncertainties—factors that often deter companies from embracing more ambitious green transformations. Meanwhile, Sun et al. (2022) point out that these difficulties commonly drive firms toward end‐of‐pipe measures, which may ensure swift regulatory compliance but fail to confront deeper ecological challenges. Taken together, these insights reveal that, although stakeholder pressure can spark interest in greener strategies, real‐world constraints in manufacturing often limit the adoption of genuinely transformative, sustainable practices.

The manufacturing sector's comparatively weaker response to stakeholder pressure on green innovation can be attributed to several interrelated factors. First, manufacturers often emphasize cutting costs and improving operational efficiency, leaving fewer resources and less priority for eco‐friendly initiatives. Unlike their counterparts in less resource‐intensive sectors such as technology or services, where stakeholder demands can more easily catalyze green strategies, manufacturers face substantial investments and lengthy payback periods for implementing environmental innovations, making them less inclined to undertake such changes without a clear and immediate payoff. Additionally, strict regulations in the manufacturing industry mean that many firms believe meeting these mandated standards already satisfies stakeholder expectations, reducing the urgency for further innovation. Beyond compliance, the emphasis among manufacturing stakeholders tends to center on traditional metrics like cost control, quality, and delivery times, relegating environmental considerations to a lower priority. By contrast, sectors that engage closely with consumers, who are increasingly aware of and concerned about sustainability issues, may find that going above and beyond minimum standards confers a competitive advantage, thus magnifying the influence of stakeholder pressure on their green innovation efforts.

Manufacturing firms can strengthen the link between stakeholder pressure and green innovation by focusing first on what stakeholders want and need. Understanding these expectations—whether they come from customers, suppliers, investors, or local communities—is essential. Firms should regularly check in through surveys, open discussions, and collaborative forums to pinpoint environmental priorities and ensure these insights guide their green innovation plans. By being transparent—clearly sharing environmental goals, reporting progress using well‐regarded standards, and openly discussing achievements—companies can build trust and credibility. Further, involving stakeholders directly in the innovation process, for example by inviting customers to help design eco‐friendly products or organizing community events centered on environmental improvements, can enrich both the innovation itself and relationships with those who have a vested interest. Collectively, these efforts turn the pressure from stakeholders into a positive force that can drive meaningful and lasting progress in sustainability.

One of the noteworthy insights from the study is that the connection between stakeholder pressure and green innovation in China is frequently regarded as less robust than in other parts of the world. The academic conversation on this matter is far from settled, presenting both supportive and dissenting viewpoints. On one hand, some researchers point out that stakeholder influence on environmental issues within China remains comparatively restrained. For instance, Yu et al. (2017) noted that although stakeholder engagement in ecological concerns has been on the rise, its impact still falls short of what is commonly seen in Western economies.

Along similar lines, Yang et al. (2022) emphasized that the Chinese manufacturing sector lags behind global frontrunners like the United States, Japan, and Germany when it comes to green innovation. Their findings suggest that despite ongoing investment in innovation, many opportunities remain underutilized, and China's manufacturing still occupies a lower‐tier position in global value chains. Moreover, Luo et al. (2023) highlighted that the beneficial effects of urban digital economies on China's green innovation are not uniform nationwide. They found that such influence tends to be curbed by factors like city size, with more significant progress in the advanced eastern regions and comparatively weaker advances in the central and western parts of the country.

Conversely, other scholars argue that stakeholder pressure on CSR and environmental performance has been intensifying in China. Tian et al. (2015) noted that with rapid economic growth since the open‐door policy of the 1970s, concerns over environmental degradation and social instability have heightened, leading to increased stakeholder pressure. Wing‐Hung Lo et al. (2010) also emphasized that various stakeholders, including central government officials, have become more vocal in demanding better environmental performance from industrial enterprises, recognizing the potential for pollution to trigger social unrest.

These contrasting perspectives reveal a complex and evolving picture. While stakeholder pressure and green innovation in China face significant challenges, there is a clear upward trajectory in stakeholder engagement and awareness of environmental issues. However, regional and sectoral disparities, as well as structural limitations, highlight the need for targeted strategies to strengthen the link between stakeholder pressure and green innovation, ensuring a more balanced and impactful transition toward sustainability.

## 6 Conclusion and Future Directions

This study provides critical insights into the relationship between stakeholder pressure and green innovation, emphasizing the moderating effects of industry and country contexts. While the findings reveal a generally positive and moderate relationship, significant variations exist, particularly in the manufacturing sector and in China, where structural and contextual challenges often hinder the full realization of green innovation. However, the growing awareness of environmental issues and increased stakeholder engagement signal a promising trajectory for sustainability efforts, highlighting the importance of tailored strategies to address regional and sectoral disparities.

This study is not without its limitations. First, relying primarily on secondary data may inadvertently exclude valuable, unpublished, or region‐specific research, potentially introducing publication bias. Second, the current focus on the manufacturing sector in China narrows the scope of the findings, limiting their applicability to other sectors and regions. Third, the cross‐sectional nature of the included studies prevents an examination of temporal trends or causal dynamics between stakeholder pressure and green innovation. Additionally, synthesizing studies with varying methodologies and quality standards may have introduced heterogeneity, affecting the robustness of the conclusions.

Future research should address these limitations by incorporating primary data collection and expanding the geographical and sectoral focus to include underrepresented industries and regions. Longitudinal studies could offer richer insights into how stakeholder pressures and green innovation evolve over time. Examining the influence of cultural, organizational, and policy‐related factors may also unveil more nuanced drivers and barriers to implementing sustainable practices. Employing more rigorous meta‐analytic techniques and pursuing sector‐specific investigations, particularly in areas that have not been thoroughly explored, can further refine our understanding of sustainable innovation across diverse contexts.

## Author Contributions

**Hasan Emin Gurler**: conceptualization, investigation, writing – original draft, methodology, software, data curation, resources, formal analysis, validation. **Ahmet Kaya**: investigation, validation, software, writing – original draft, conceptualization, resources. **Ayşe Nur Soysal Bilmiş**: investigation, validation, data curation, writing – original draft, resources. **Yakup Durmaz**: conceptualization, funding acquisition, supervision, project administration, resources, visualization, writing – review and editing.

## Ethics Statement

This study does not involve human participants, animal subjects, or sensitive personal data and therefore did not require ethical approval.

## Consent

The authors have nothing to report.

## Conflicts of Interest

The authors declare no conflicts of interest.

## Peer Review

The peer review history for this article is available at https://publons.com/publon/10.1002/brb3.70840

## Data Availability

Data supporting the findings of this study can be obtained from the corresponding author upon reasonable request.
